# Conceptual Frameworks and Practice on Nano-Micro Pore Structure Design of Bio-Aerogels

**DOI:** 10.3390/gels12100886

**Published:** 2026-10-01

**Authors:** Shuai Yuan, Jiaren Yang, Kao Wu, Hong Qian, Xi Chen, Ying Kuang, Fatang Jiang, Guangyuan Yang, Fan Zhu, Saffa Riffat

**Affiliations:** 1 School of Life and Health Sciences, Hubei University of Technology, Wuhan 430068, China; arbeit19960413@163.com (S.Y.); yang506067041@163.com (J.Y.); wukao@mail.hbut.edu.cn (K.W.); lazywawa@163.com (Y.K.); 2 School of Nursing and Health Management, Wuhan Donghu University, Wuhan 430212, China; qxueyuki@163.com; 3 WuHan LiCheng Biotechnology Co., Ltd., Wuhan 430205, China; 13297084676@163.com; 4 China Tobacco Hubei Industrial of Cigarette Material Co., Ltd., Wuhan 430051, China; 5 School of Chemical Sciences, University of Auckland, Auckland 92019, New Zealand; f.zhu@auckland.ac.nz; 6 Faculty of Engineering, University of Nottingham, University Park, Nottingham NG72RD, UK; saffa.riffat@nottingham.ac.uk

**Keywords:** biomass, aerogel, pore structure design, theory

## Abstract

Bio-aerogels have garnered significant attention due to their environmental friendliness and potential for exceptional performance. The pore structure of bio-aerogels includes both pore size and distribution, which significantly determine their functional behaviors, such as filtration, thermal insulation, and sound absorption. This review proposes several conceptual frameworks regarding the design of the pore structure of bio-aerogels, some of which are validated through practical cases. The ice crystal pore-making conceptual framework approach involves controlling the growth of ice crystals to achieve a pre-designed pore structure. The space occupying pore-making conceptual framework is the proactive addition of nano materials to occupy space prior to the formation of ice crystals, facilitating the design of the aerogel’s nanoscale pore structure. The conceptual framework of blocks building involves assembling multiple aerogels to create a composite aerogel with a multi-level pore structure. These theoretical considerations contribute to the development of aerogel structural design theory to obtain aerogels with different micro-nano structures.

## 1. Introduction

Aerogel, an ultra-light solid material distinguished by its porous network structure, is characterized by low density, low thermal conductivity, high surface area, high porosity, excellent mechanical properties, flame retardancy, low refractive index, and low dielectric constant [1,2,3,4]. Initially, some researchers contended that only materials produced through supercritical drying technology could be classified as aerogels, imposing stringent criteria on these materials’ pore size and porosity. However, as aerogel materials have evolved, the definition has shifted to emphasize material properties over the preparation technology employed [5,6,7,8]. Any three-dimensional porous materials characterized by low density, with pore sizes in the micron and nanoscale range can be designated as aerogels.

Kistler first reported aerogels in 1931 [9]. High-performance aerogels composed of silica and its derivatives, as well as metals, metal oxides, and semiconductor nanoparticles (NPs), have garnered considerable attention in the field of aerogel materials [10,11]. However, the components required for the preparation of these inorganic aerogels are often costly and challenging to degrade, posing environmental concerns and limiting their practical applications [12,13]. With the increasing need to address environmental issues, a growing number of biomass materials—such as cellulose, alginate, chitosan, pectin, and starch—are being explored as potential raw materials for aerogel production [14,15,16]. These materials are derived from renewable sources and are typically processed via freeze-drying or supercritical drying, resulting in bio-aerogels—a subclass of aerogels that are distinguished from conventional inorganic (e.g., silica-based) or synthetic polymer aerogels by their biobased origin, inherent biodegradability, and reduced environmental footprint [17,18,19]. Bio-aerogels retain the characteristic properties of aerogels, such as low density, high porosity, and high specific surface area, while offering additional advantages, including feedstock renewability, biocompatibility, and ease of surface functionalization [20,21,22].

Bio-aerogels possess a remarkable microstructural characteristic: the presence of abundant hierarchical pore structures within their interior [23]. These pore structures confer significant advantages to the material, including a high specific surface area, high porosity, and low density. Serving as a crucial link between intrinsic material properties and practical functional applications, the core characteristics of the pore structure can be primarily described by two fundamental parameters: pore size and size distribution.

The pore size refers to the diameter of the channels and directly determines the fundamental physical mechanisms of material transport and interaction within aerogels. The International Union of Pure and Applied Chemistry (IUPAC) classifies pores into three categories: micropores (less than 2 nm), mesopores (2–50 nm), and macropores (greater than 50 nm). The pore size distribution characterizes the proportion of pores of varying sizes within a material, reflecting the homogeneity of the pore structure, which is crucial for determining the potential for synergistic functions.

While extensive studies have evaluated the functions and applications of biomass aerogels, a systematic reflection on pore structure design strategies spanning nano-to-micro scales remains absent. This review aims to fill this gap by proposing three conceptual design frameworks—ice crystal pore-making, space-occupying, and blocks building—which systematically classify established strategies. Although these frameworks do not introduce new physical mechanisms, they provide a cohesive design logic that integrates established strategies across various length scales and offers novel design capabilities beyond existing classifications. Specifically, the ice-crystal pore-making framework highlights the previously overlooked roles of biomass assembly structure and water activity in modulating ice growth and the resulting pore architecture. The space-occupying framework introduces an innovative design concept for nanoscale and submicron pore engineering, effectively addressing the limitations associated with ice templating at the micrometer scale. Lastly, the blocks-building framework presents a new conceptual approach for constructing multifunctional aerogels with hierarchical pore structures through the modular assembly of independently optimized units (Table 1). These capabilities are not systematically addressed in current reviews. Although quantitative models that correlate processing parameters with final pore structures have yet to be established—representing a critical direction for future research—these strategies offer a systematic foundation for rational material design, supported by practical case studies in sound insulation, thermal insulation, and filtration purification.

## 2. Ice Crystal Pore-Making Conceptual Framework

**Figure 1:**
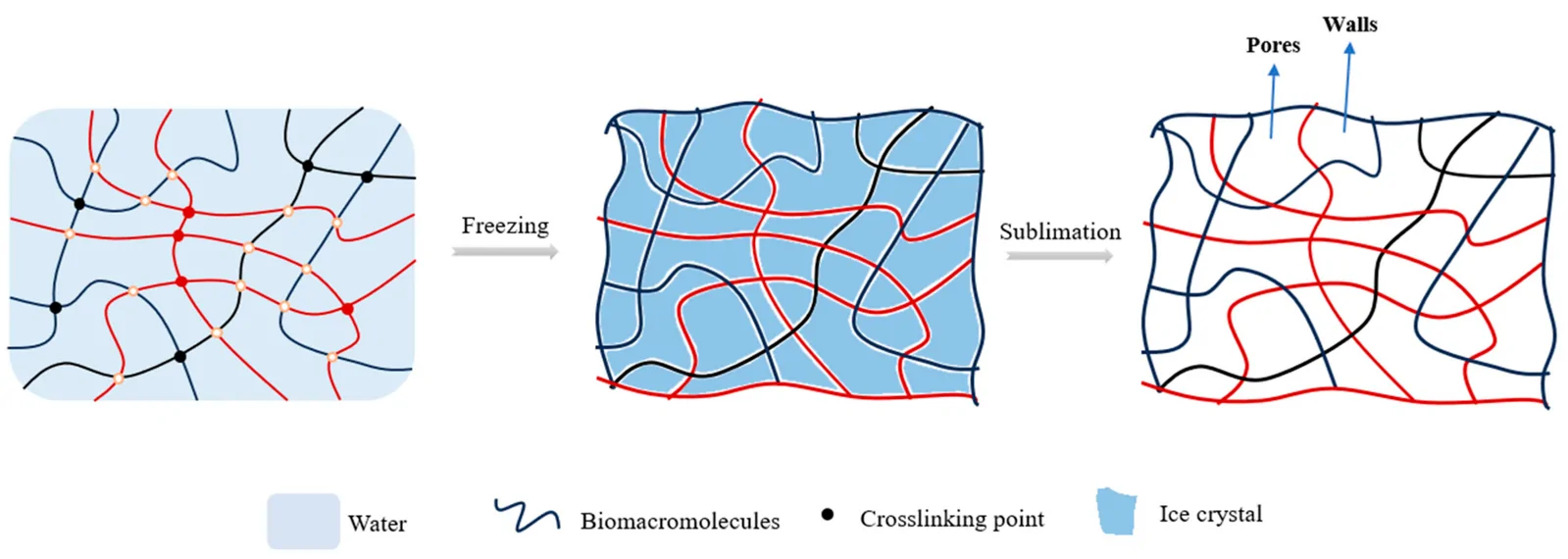
Formation mechanism of bio-aerogel porous structure based on ice crystals.

The preparation and structural formation of bio-aerogels typically involve several steps, including dispersion, gelation, freezing, and sublimation. Initially, biopolymer molecules are hydrated or dispersed in a water-based medium to prepare a precursor dispersion. This precursor dispersion is then transformed into a hydrogel by altering physical conditions or incorporating chemical cross-linking agents. Subsequently, the hydrogel is frozen, which causes the water within the gel network to solidify, resulting in the enrichment of the polymer at the ice grain boundaries [27]. Finally, the ice crystals are removed through sublimation under vacuum, yielding a pore structure in the aerogel that corresponds to the ice crystals (Figure 1). By manipulating the material components or the freezing process, one can adjust the size and distribution of the ice crystals, ultimately enabling the design of bio-aerogels with varying pore structures [28,29]. Due to the current limitations in cryogenic control technology, achieving precise control over ice crystal growth at the nanoscale remains a significant challenge. Consequently, the pore structures of biomass aerogels designed using ice-templating theory are predominantly at the micrometer scale.

### 2.1. Physical Mechanisms of Ice Crystal

According to classical nucleation theory, water molecules in a liquid initially form crystal nuclei during the crystallization process, which subsequently grow into ice crystals [30]. It is crucial to understand that ice crystal nucleation and growth occur simultaneously and are interrelated processes [31]. Although the freezing crystallization of water requires supercooling, excessive supercooling can hinder crystal growth. At elevated levels of supercooling, the nucleation rate exceeds the crystal growth rate, creating conditions that favor the occurrence of water molecule nucleation events. This results in the formation of numerous small ice crystals and a uniform pore structure [32].

Altering freezing conditions can significantly influence the structural design of aerogels formed by ice crystals (Table 2). Significant differences in ice crystal size were observed in the precursor dispersion solution at varying freezing temperatures. Smaller ice crystals formed at higher degrees of supercooling, resulting in a corresponding shift in their pore size. The pore structure size of aerogels prepared under different freezing conditions exhibited trends similar to those of the ice crystals [33]. The rate and direction of ambient cooling impact the size of the ice crystals that develop; rapid cooling leads to a greater number of smaller crystals, whereas slow cooling results in the appearance of larger crystals. A high cooling rate encourages the formation of more ice crystals, minimizes the agglomeration of biomass molecules, and enhances the porosity of bio-aerogels [34].

During the growth of ice crystals, a temperature field is applied to facilitate the phase transition from the dispersion medium (water) to ice. By controlling the directionality and intensity of the temperature field, the growth direction and size distribution of the resulting ice can be regulated, thereby allowing for the design of the aerogel pore structure [40]. Unidirectional freezing involves cooling a single surface of the solution, which results in ice crystals that are aligned in one direction and a uniformly distributed pore size [41]. In contrast, bidirectional or multi-directional freezing employs multiple temperature fields superimposed on the solution, leading to continuously varying heat exchange rates and resulting in diverse final pore size distributions. In one-way freezing, the temperature gradient is distributed horizontally, producing a parallel arrangement of the material matrix and an interconnected porous structure. Conversely, in bidirectional freezing, temperature gradients develop in both horizontal and vertical directions, causing the ice and the polymer composite matrix to grow simultaneously in two directions, thereby forming a distinctive bridge-like structure [30]. Different pore structures are generated in aerogel samples with the same components when subjected to unidirectional versus bidirectional freezing.

Although achieving precise nanoscale control of ice crystal growth remains challenging, several promising strategies have emerged. These include the introduction of exogenous nanoscale nucleating agents, such as cellulose nanocrystals and chitin nanofibrils, to promote heterogeneous nucleation and increase nucleation density [42,43]. Additionally, ultrafast freezing techniques, such as spray freezing and cryogenic jets, can achieve ultrahigh supercooling, effectively limiting ice crystal growth to the submicron or nanoscale. Furthermore, the combination of the ice-templating strategy with the space-occupying strategy—such as pre-embedding nano-emulsion droplets to confine ice growth—presents a potential hybrid approach for nanoscale pore engineering [44,45]. These pathways merit further experimental investigation.

### 2.2. The Influence of Biomacromolecule Assembly

The molecular structural complexity and self-assembly capabilities of biomass components collectively determine the hierarchical process of biomass aerogel formation, which spans from molecular assembly to the molding of macroscopic porous structures. Specifically, biomass polymers, such as proteins and polysaccharides, undergo self-assembly or co-assembly through non-covalent interactions and covalent bonds, resulting in the formation of nanoscale primary structures, including fibers, spheres, and lamellae. These primary structures further aggregate, entangle, and cross-link to construct a three-dimensional gel network, known as a hydrogel [46]. The structural characteristics of this network, including network density, chain rigidity, and distribution of cross-linking points, directly influence the mechanical properties and spatial configurations of the assemblies in the precursor dispersion [47,48]. During the subsequent freezing process, dynamic interactions occur between the pre-formed gel network and the growth of ice crystals [49]. The strength, toughness, and spatial scale of the gel network itself determine its ability to resist and constrain the growth of ice crystals, thereby exerting a decisive influence on the final pore structure of the aerogel.

Fibrous networks, such as cellulose, chitin nanofibrils, and Ca^2+^-crosslinked alginate, form three-dimensional structures through hydrogen bonding and entanglement among rigid chains [50,51]. This arrangement restricts ice nucleation and growth, promoting the formation of fine and uniform ice crystals. Particulate and spherical aggregated structures, including globular proteins like soy protein and whey protein, as well as starch granules, form submicron aggregates under specific induction conditions [52,53]. These aggregates construct gel networks via hydrophobic interactions and disulfide bonds. Under the compression of ice crystals, these networks undergo plastic deformation, with aggregates migrating toward and enriching at the boundaries of the ice crystals, resulting in aerogels characterized by thick pore walls and dense skeletal structures [54,55]. Moreover, entangled and random coil structures, such as xanthan gum and hyaluronic acid, rely on physical entanglement to maintain dynamic networks. In these networks, chain segments readily migrate with advancing ice fronts, offering limited restriction to ice crystal growth and tending to form honeycomb-like macropores [6].

### 2.3. Water Activity

In structural design, the influence of biomass matrices on the water state within precursor solutions is frequently underestimated. Water activity (Aw) refers to the ratio of the vapor pressure of water in a substance to the saturation vapor pressure of pure water at the same temperature [56]:

Aw = P/P_0_

P is the partial pressure of water vapor when a certain substance reaches equilibrium in a closed container; P_0_ is the saturated vapor pressure of pure water at the same temperature. A landmark contribution by Koop et al. established that homogeneous ice nucleation from supercooled aqueous solutions is independent of the nature of the solute and depends solely on the water activity of the solution [56]. This finding, supported by systematic experiments across diverse solutes—including salts, organic molecules, and polymers—reveals a universal relationship: the ice nucleation temperature is determined by water activity rather than by solute identity. In essence, water activity serves as a measure of ‘free water’—the fraction of water molecules that are not tightly bound to solutes or polymer matrices. As water activity decreases, the chemical potential of water is reduced, necessitating a greater degree of supercooling (i.e., lower temperature) to overcome the thermodynamic barrier for ice nucleation.

Water activity serves as a fundamental parameter that quantifies the availability of water in the system, illustrating the strength of interactions between water molecules and biomass molecules [57,58]. When biomass polymers are dissolved or dispersed in water, they participate in various interactions with water molecules, effectively ‘binding’ a portion of these molecules and consequently diminishing their freedom to transition from the liquid phase to the gas phase.

The impact of water activity on the freezing process is primarily manifested through alterations in the thermodynamics and kinetics of water phase transitions. Specifically, lower water activity diminishes the tendency of water molecules to escape from the liquid phase, necessitating a greater energy barrier for the liquid-to-solid transition and resulting in a lower freezing point [56,59,60]. Consequently, a lower temperature is required to achieve the necessary supercooling for nucleation. At higher supercooling, the nucleation rate exceeds the crystal growth rate, leading to a higher density of ice nuclei. Simultaneously, the elevated viscosity and reduced molecular diffusion in the freeze-concentrated phase limit the growth of each ice crystal. The combined effect is the formation of numerous small ice crystals with a narrow size distribution [58]. After sublimation, these ice crystals yield smaller pores with a narrower pore-size distribution.

The ice crystals formed during freezing serve as templates that directly determine the pore architecture of the aerogel after sublimation [60]. Therefore, the control over ice nucleation and growth mediated by water activity provides a thermodynamically grounded pathway for pore structure engineering. Specifically, Lower Aw (e.g., achieved through higher polymer concentration or stronger water-binding capacity) results in smaller ice crystals, leading to smaller pores with a narrower distribution. Higher Aw (approaching that of pure water) results in fewer, larger ice crystals, resulting in larger pores with a broader distribution [61]. This Aw-based framework elucidates why different biomass feedstocks—with distinct molecular structures and water-binding capacities—yield varying pore structures even under identical freezing conditions [58,62]. For instance, polysaccharides with higher hydrophilicity or more extended chain conformations typically exhibit greater reductions in Aw and consequently produce finer pore structures.

## 3. Space-Occupying Conceptual Framework

Bio-aerogels prepared through freeze-drying primarily regulate ice crystal growth by manipulating freezing parameters and biomass components to achieve a designed pore structure. However, current limitations in controlling nanoscale ice crystals restrict the applicability of the ice crystal pore-making conceptual framework to nanometer-level structural design [32]. We propose a space occupying pore-making conceptual framework, wherein space-occupying materials are introduced prior to the solidification of the dispersion medium to occupy specific spatial positions. This approach ensures that the resulting aerogel’s pore structure is independent of ice crystals. During the freeze-drying process, both the space-occupying materials and the dispersion medium are subsequently removed. The incorporation of nano space-occupying substances allows the pore structure of aerogels to exceed the size limitations imposed by ice crystals during freezing, thereby enhancing design efficiency in terms of pore size and size distribution, and facilitating pore structure design at the nanoscale. In the following subsections, we will discuss three different space-occupying modes to achieve this objective.

### 3.1. Sodium Polyacrylate (SAP)

**Figure 2:**
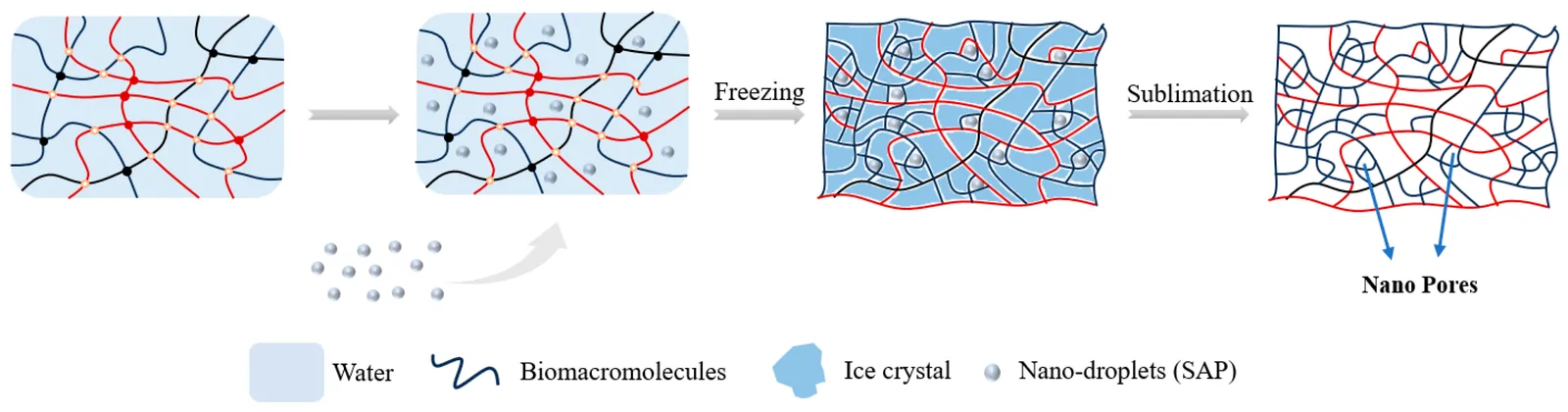
Mechanism diagram of Space occupying pore-making conceptual framework (SAP).

SAP is a superabsorbent polymer known for its remarkable water-absorbing capacity. Its molecular structure features numerous sodium carboxylate groups (-COONa) that form hydrogen bonds with water molecules, allowing SAP to absorb and retain hundreds or even thousands of times its own weight in water. By regulating the duration of water absorption or hydration, nano-SAP particles can achieve limited water absorption (ranging from several to dozens of times) to prevent excessive swelling. The partially absorbed SAP forms a hydrogel layer comprising hydrophilic polymer chains on its outer surface, which can be conceptualized as nano-droplets. Before freezing, these nanodroplets were introduced into the precursor solution as space-occupying material. During the subsequent freezing process, the confined water within the SAP hydrogel droplets, along with the free water in the external dispersion medium, solidifies into ice crystals [63]. Crucially, the size of the SAP droplets spatially constrains the growth of ice crystals within each droplet, limiting them to a smaller scale. In the freeze-drying process, the ice crystals—both within the droplets and in the external medium—are removed via sublimation, creating void spaces that become the pores. The SAP polymer network itself does not sublime; upon dehydration, it undergoes collapse and densification, leaving behind a dense polymer residue that forms part of the pore wall material. Therefore, the pores obtained from SAP are embedded in the pore walls and can ideally reach the nanoscale. The final pore structure reflects the initial size of the SAP hydrogel microdroplets (Figure 2).

The process of SAP occupying space for pore formation is divided into three key stages, with a primary focus on maintaining the independence and stability of the space-occupying material. In the first stage, SAP forms nanodroplets through limited water absorption, which are then introduced into the precursor dispersion. In the second stage, following the introduction of the nanodroplets, rapid freezing is applied to prevent further swelling of the SAP within the dispersion, thereby preserving the nanoscale size of the space-occupying material and avoiding interference. During the freezing process, as the free water phase in the precursor dispersion solidifies into large ice crystals, the nanodroplets are simultaneously frozen into nanoscale ice crystals. Furthermore, the introduction of nanodroplets reduces the water activity of the precursor dispersion, which in turn decreases the overall size of the formed ice crystals.

Despite its advantages, the SAP-based space-occupying strategy faces several critical challenges. First, the homogeneous dispersion of SAP nanogels in the biopolymer matrix is hindered by their strong hydrophilicity and tendency for aggregation, especially at high concentrations [64]. Second, the chemical compatibility between SAP and biomass precursors requires careful optimization, as incompatible surface chemistries may lead to phase separation or impaired gel network formation. Third, excessive addition of SAP may compromise the mechanical performance of the resulting aerogels, as SAP nanogels leave voids after sublimation, potentially reducing the load-bearing capacity of the scaffold. Thus, balancing pore design efficacy with mechanical integrity remains a key consideration for practical applications.

Although ice templating within superabsorbent polymer (SAP)-containing hydrogels has been demonstrated, direct validation of pore replication in biomass aerogels using SAP as a space-occupying template remains lacking. Consequently, the achievable pore size range presented in Table 1 should be regarded as an estimate based on the dimensions of SAP hydrogel droplets, serving as a design target rather than an experimentally confirmed value.

### 3.2. Nano-Sublimable Substances (Iodine)

**Figure 3:**
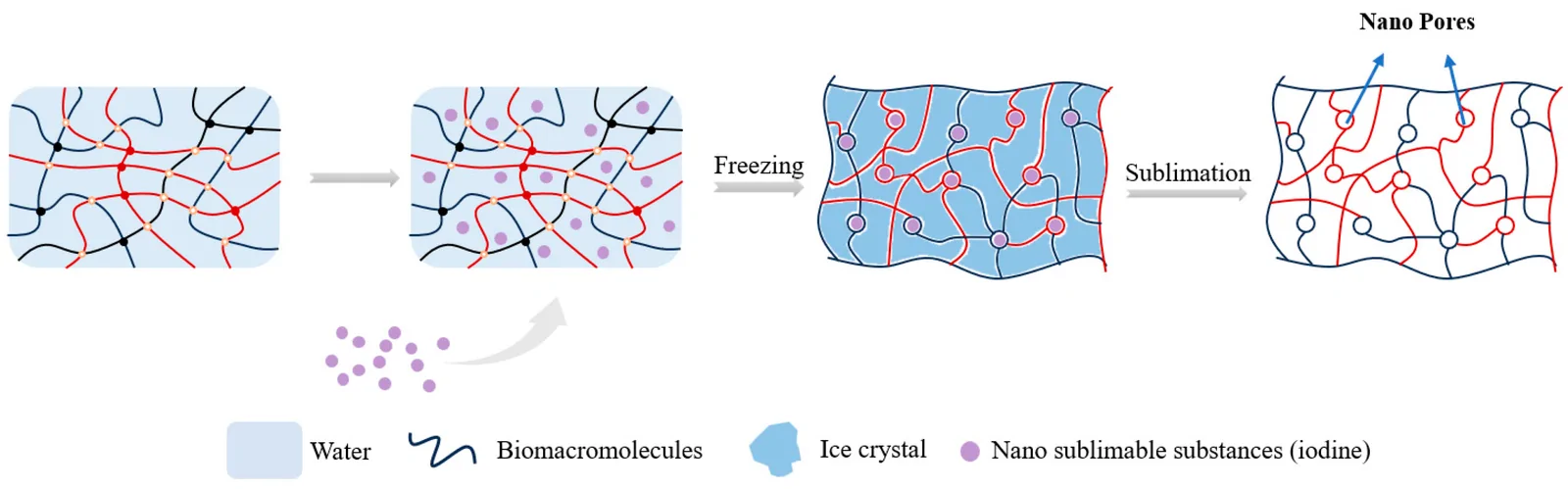
Mechanism diagram of Space occupying pore-making conceptual framework (Nano-sublimable substances: iodine).

The underlying principle of the iodine space-occupying approach is rooted in the well-established sacrificial template strategy, wherein the dimensions of the final pores are dictated by the size of the space-occupying particles. In this context, the pore size of the resulting aerogel is anticipated to correspond directly to the initial size of the iodine nanoparticles, provided that (i) the iodine crystals are uniformly dispersed in the precursor solution without significant aggregation, and (ii) the iodine crystals maintain their integrity during the freezing process without dissolution or recrystallization [65]. This principle of size correspondence has been demonstrated across various template systems, where systematic variations in template particle size lead to predictable alterations in pore dimensions. For iodine-treated precursors, research has indicated that iodine can function as templates and activators for pore development (Figure 3).

It is important to acknowledge that the spatial distribution of iodine nanoparticles may be altered during the freezing process. As ice crystals grow, the advancing solidification front exerts a pushing effect on suspended particles, potentially causing their redistribution toward grain boundaries or inter-dendritic regions. This phenomenon, known as particle segregation or ice-front pushing, is well-documented in freeze-casting processes and could lead to a non-uniform pore distribution if the iodine nanoparticles are redistributed during freezing. Additionally, the high concentration of iodine nanoparticles in localized regions may promote aggregation, further compromising pore uniformity. Mitigation strategies include employing rapid freezing rates to minimize particle migration and incorporating dispersants or stabilizers to prevent nanoparticle aggregation.

The iodine space-occupying approach is presently in the conceptual stage and is pending experimental validation. Its theoretical foundation is strongly based on the phase behavior of iodine: under typical lyophilization vacuum conditions (1–50 Pa), the sublimation temperature significantly decreases from the triple-point value of 113.7 °C (12.1 kPa), allowing for its removal during secondary drying at temperatures ranging from 30 to 50 °C. This compatibility with standard equipment indicates technical feasibility; however, challenges remain concerning the uniform dispersion of nano-iodine and the prevention of aggregation during the freezing process [66].

Despite its conceptual elegance, the iodine space-occupying approach encounters several practical challenges that must be addressed for successful implementation. First, the large-scale preparation of monodisperse nano-iodine crystals with controlled size and narrow size distribution remains technically demanding. Second, maintaining the stable dispersion of hydrophobic iodine nanoparticles in aqueous biomass precursor solutions necessitates the use of appropriate surfactants or stabilizers, which may introduce additional components that could interfere with gelation or leave residues. Third, while the sublimation removal step is theoretically feasible, it adds complexity and energy consumption to the manufacturing process, potentially increasing production costs. Fourth, the use of iodine—a corrosive and volatile substance—raises safety and equipment compatibility concerns, particularly for large-scale operations. These limitations indicate that the iodine templating approach may be best suited for specialized applications where precise nanoscale pore control justifies the additional processing complexity, rather than for general-purpose, large-scale production.

The iodine approach remains at a conceptual stage. While iodine has been used as a pore-making agent in porous carbon [25], its application as a space-occupying material for pore replication in biomass aerogels has not yet been experimentally demonstrated. The pore size range given in Table 1 is therefore an estimate based on the size of iodine nanoparticles, and requires experimental verification.

### 3.3. Nanoscale Bubbles

**Figure 4:**
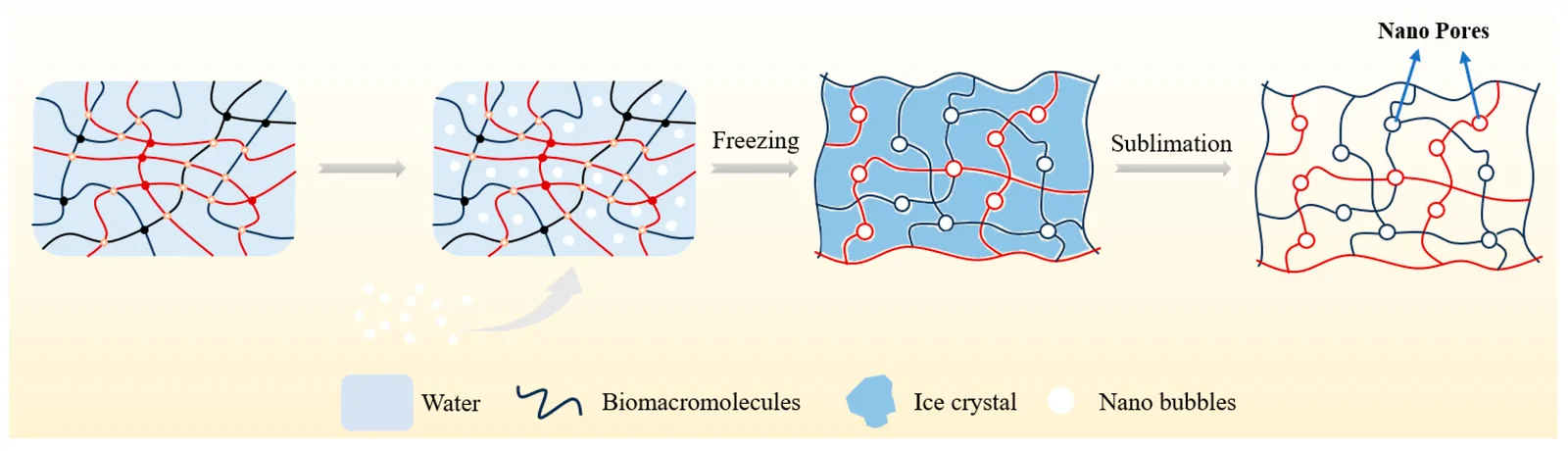
Mechanism diagram of Space occupying pore-making conceptual framework.

In conventional understanding, bubbles in liquids are transient, unstable, and prone to coalescence or dissolution. However, nanobubbles exhibit exceptional stability due to the balance between internal Laplace pressure and external electrostatic repulsion resulting from surface charges [67]. This unique characteristic qualifies them as “space-occupying substances.” The core principle of their pore-forming process involves bypassing the “water-ice” phase transition step for pore creation, instead directly utilizing a pre-existing, stable gas–liquid interface to define the final pore structure (Figure 4).

The introduction of nanobubbles is a prerequisite for achieving occupation, which can be categorized into two approaches: exogenous introduction and endogenous generation. Exogenous introduction involves incorporating well-monodispersed nanobubbles into biomass precursor dispersions through specific techniques (e.g., solvent exchange, ultrasonic cavitation, microfluidics). Following the introduction of nanobubbles, the biomass precursor initially formed a continuous dispersion phase around the bubbles. At this stage, the spatial position, size, and distribution of the nanobubbles had already established the fundamental framework for the future aerogel pore structure [68].

As the system began to freeze, ice crystals nucleated and grew from the supercooled free water phase. Similar to how biomass particles are displaced, the advancing fronts of the ice crystals also displaced the pre-existing nanobubbles. During the growth of the ice crystals, the nanobubbles served as space-occupying substances, physically obstructing the ice crystals from extending into their occupied spaces. Consequently, the ice crystals were compelled to grow around the bubbles, compressing and concentrating both the bubbles and the surrounding biomass network at the boundaries between the ice crystals. In an ideal rapid freezing scenario, the growth rate of ice crystals is sufficiently rapid that nanobubbles cannot escape through diffusion or coalescence. However, the nanobubbles themselves are not removed by sublimation, as they are gaseous rather than solid. Instead, the gas within the nanobubbles is released through diffusion and escapes via the interconnected pore network under vacuum conditions, or it may dissolve and diffuse away, leaving behind voids that correspond to their original spatial positions. This distinction between gas diffusion/escape and ice sublimation is essential for a physically consistent description.

The spaces originally occupied by the nanobubbles are thus preserved, resulting in a series of spherical or near-spherical pores that closely correspond to the initial size and morphology of the nanobubbles. In comparison to other space-occupying materials, bubbles are environmentally friendly and pollution-free, aligning with the principles of green manufacturing [69].

However, it is essential to emphasize that this concept remains largely theoretical, and its practical feasibility faces significant challenges. First, achieving long-term and uniform dispersion of nanobubbles in complex biomass polysaccharide solutions presents a primary hurdle. Second, the growth of ice crystals during the freezing process may displace or disrupt the nanobubbles, thereby diminishing their space-occupying effect [70].

The nanobubble approach is currently in an early exploratory stage. Preliminary experimental evidence supports the use of nanobubbles for constructing hierarchical pores within cryogens made from chitosan and regenerated silk fibroin [26], suggesting their potential as effective pore-forming agents in biomass systems. However, there is still a lack of direct validation for pore replication in biomass aerogels, and the quantitative relationship between nanobubble size and the resulting pore dimensions has yet to be established. Consequently, the achievable range of pore sizes remains undetermined. The mechanism outlined herein, which is based on the established stability of nanobubbles and their interaction with ice fronts, should be viewed as a promising research direction that necessitates further experimental verification rather than a fully validated strategy.

## 4. Blocks-Building Conceptual Framework

The classical sol–gel method is a continuous and irreversible chemical process. The gradients of its chemical reactions, including hydration and polycondensation, are difficult to control precisely, which hinders the spatial design of distinctly different microstructural domains. Once gelation commences, the structure is essentially fixed and cannot undergo significant non-uniform structural adjustments. This limitation restricts the diversity and customizability of properties. The macroscopic morphology and microscopic structure of aerogel materials are simultaneously formed and locked within the same chemical reaction system, making the achievement of any complex internal heterogeneous structure extremely challenging.

**Figure 5:**
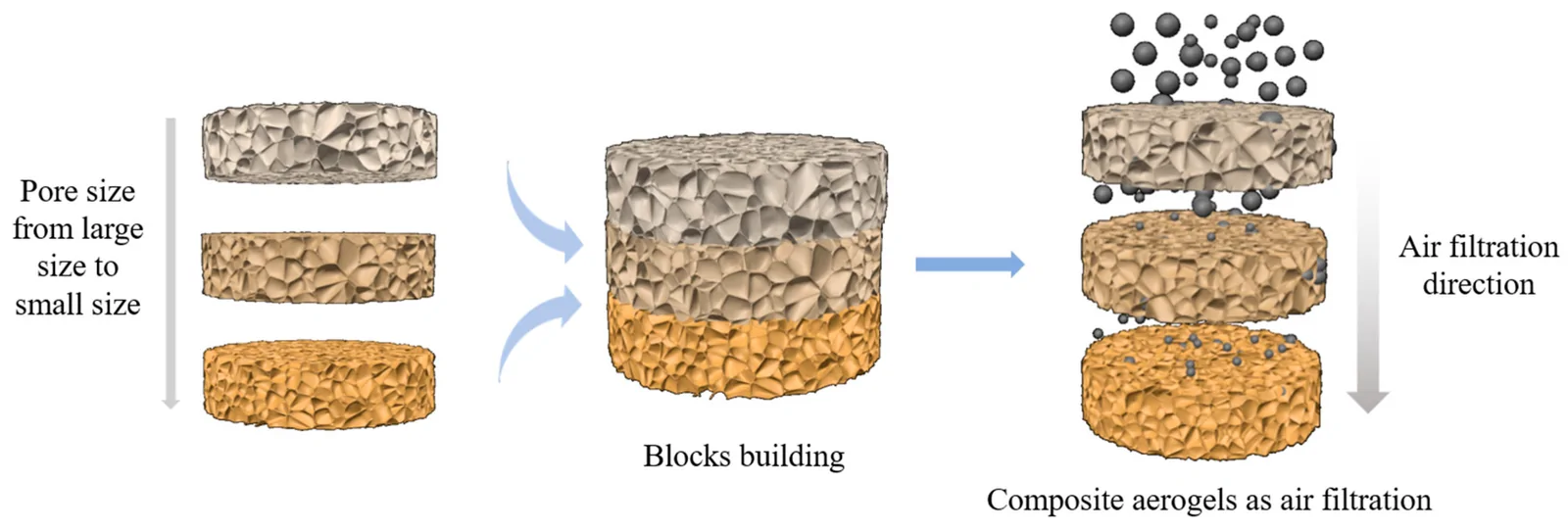
Schematic illustration of the blocks-building strategy for designing hierarchical aerogels for filtration.

Each independent aerogel unit, characterized by a uniform microstructure, is considered a “building block” or “module.” Each module can independently optimize its performance; for instance, one module may perform adsorption while another provides mechanical strength (Figure 5). By employing various assembly methods, such as series connection and layer-by-layer stacking, we can achieve the design of aerogel structures with multi-level complex configurations [71].

Based on the aforementioned concepts, the design pathway for composite aerogels becomes evident. In accordance with the requirements of the target application, the desired multifunctional and multilevel structures are decomposed into several single-function and single-structure requirements. For each requirement, the most optimized structural unit of the aerogel is designed and fabricated. The spatial arrangement of these units—whether in series, stacking, or composite form—is determined, leading to the creation of an ‘assembly diagram.’ Subsequently, these units are integrated into a macroscopic whole through physical or chemical methods. When employing bio-aerogels as filters for particle filtration, a design strategy that transitions from larger pores to smaller pores can be effectively implemented. The larger openings on the inflow side mitigate the accumulation of intercepted particles, thereby decreasing the risk of channel clogging and enhancing the circulation of the filtered fluid. Conversely, the smaller pores on the outflow side capture particulate pollutants incrementally, thereby improving the overall interception rate of pollutants (Figure 5). Compared to single-structure aerogels, composite aerogels demonstrate superior interception capabilities and excellent gas flow properties [72,73].

The blocks-building theory has transformed the traditional ‘top-down’ integrated manufacturing paradigm in aerogel structural design into a ‘bottom-up’ modular assembly paradigm, effectively addressing the challenges associated with complex structure fabrication. This approach allows aerogels to transcend the limitations of being a fixed, homogeneous block of material; instead, they can be viewed as systems capable of ‘constructing’ their internal structure and functionality through various ‘blocks-building’—that is, the combinatorial schemes of base units.

The blocks-building strategy, while offering structural versatility, presents several significant challenges. A primary concern is the interfacial adhesion between adjacent modules; weak adhesion can compromise structural integrity under mechanical loading. Chemical or physical crosslinking at interfaces may enhance adhesion, but it may also alter the local pore structures. Moreover, module stacking inevitably increases the interface density, which may become preferential sites for defect formation (e.g., microcracks, delamination), adversely affecting both mechanical robustness and filtration/thermal insulation performance. Therefore, future efforts should concentrate on engineering robust interfaces through tailored surface chemistry and developing assembly methods that minimize defect generation.

## 5. Practice Case

### 5.1. Filtration and Purification

With the rapid development of industry, the sharp increase in greenhouse gases and other air pollutants has rendered green and efficient air purification an urgent issue. In contrast to non-environmentally friendly materials such as polyacrylonitrile or amines, biomass aerogels present a highly promising alternative for air filtration due to their continuous three-dimensional network structure, ultra-low density, high specific surface area, significant porosity, and interconnected networks that enhance gas–solid interactions [74,75]. The filtration performance of aerogels arises from the synergistic interplay of multiple structural parameters, including pore size, porosity, connectivity, and tortuosity. Pore size is a primary determinant of filtration precision, as smaller and more uniform pores enhance the interception efficiency of submicron particles [76]. Porosity affects dust-holding capacity and air permeability; highly porous materials can accommodate a greater number of particles, thereby extending their service life [77]. Connectivity influences the ease of fluid transport through the material; vertically aligned channels, created through directional freezing, significantly reduce tortuosity and greatly enhance filtration flux [78]. Tortuosity reflects the complexity of the pore network: highly tortuous pathways increase the probability of particle-wall collisions and improve filtration efficiency, although they also elevate flow resistance. Consequently, an ideal filtration aerogel should achieve a balance between high porosity and macroporous channels (to minimize resistance) and a highly tortuous mesoporous network (to maximize precision). KGM-based aerogels have been employed in air filtration research, achieving a filtration efficiency of 93.54% for particulate matter (≥0.3 μm), with a filtration resistance of 29 Pa and an air permeability of 271.42 L/s·m^2^. This demonstrates the significant potential of biomass aerogels in air purification applications [79]. Additionally, a lignocellulosic aerogel with exceptional overall performance has been developed, utilizing tourmaline (TP) and its abundant porous structure to release negative oxygen ions (NOI), thereby enhancing air purification capabilities [80].

**Figure 6:**
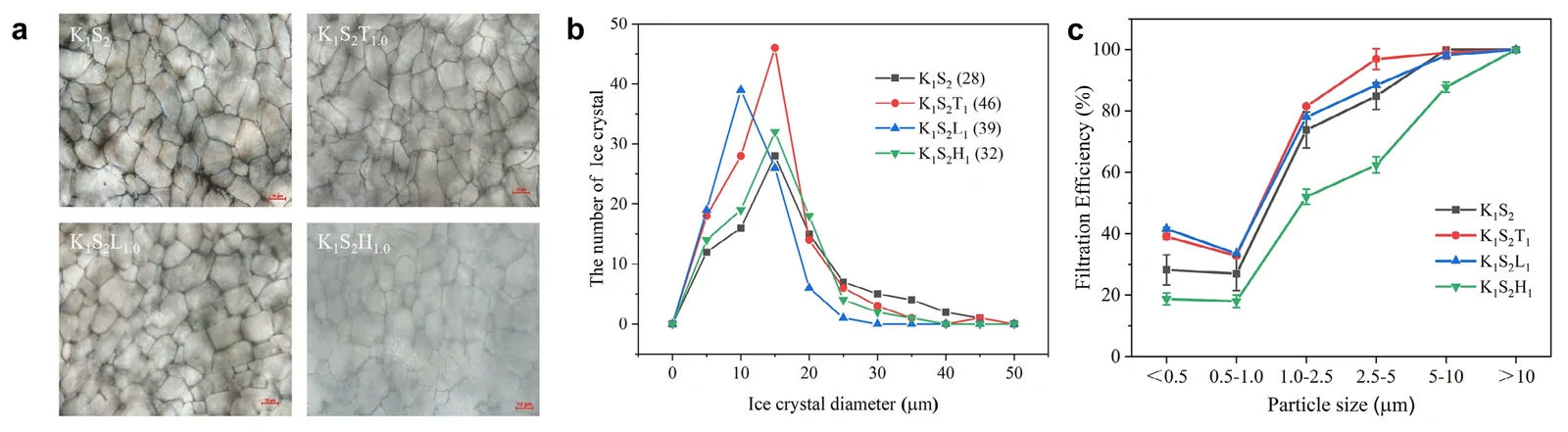
(**a**,**b**) Morphology and size distribution of ice crystals of KGM/starch sol with different antifreeze protectant additions during freezing. (**c**) Filtration efficiency of KGM/starch aerogel with different antifreeze protectant additions [78]. Copyright 2025, Wiley-VCH.

The ice crystal pore-making theory enables the effective design of pore size and distribution in biomass aerogels by regulating the dimensions of ice crystals during the freezing process. This regulation reduces air flow resistance and enhances filtration efficiency. Through the modification of biomass components, it was observed that the incorporation of curdlan led to a reduction in micropore size and an increase in pore count within KGM-based aerogels, consequently enhancing filtration efficiency. KC aerogels exhibited over 80% filtration efficiency for both PM1.0 and PM2.5, with stable filtration performance and pore structure maintained after 15 filtration cycles, indicating a long service life [6]. The effects of adding three types of cryoprotectants on ice crystal growth were compared to guide the pore structure design of biomass aerogels. Among these, the addition of trehalose led to the formation of smaller ice crystals, which resulted in smaller and more uniform pore sizes in the aerogels (Figure 6a–c). This modification also contributed to the aerogels achieving the highest compressive strength, thereby effectively enhancing their air filtration performance [81].

Air purification primarily targets two categories of pollutants: particulate matter (PM) and gaseous pollutants, such as volatile organic compounds (VOCs). While smaller pore sizes can lead to higher filtration efficiency, they also increase air resistance, resulting in greater energy consumption. Therefore, the ideal structure comprises a hierarchical pore system consisting of macropores and mesopores, which can effectively capture small particles while facilitating smooth air flow. The blocks-building theory can be utilized to design biomass aerogels featuring multilevel pore structures, thereby enhancing their filtration and purification performance. An air filter constructed with multi-layered KGM-based aerogels, featuring a pore size distribution from large to small, has been designed. This design enables the KGM-based aerogel to achieve filtration performance comparable to that of commercial air filters while providing an extended purification duration. Furthermore, by either adjusting the pore size distribution or increasing the specific surface area through folded structures, the purification time can be reduced by over 50% [7]. KGM-based aerogels with varying pore size distributions were assembled into laminates to enhance their filtration performance. It was observed that the aerogel laminate, prepared by serially combining aerogel sheets with different pore size distributions (from large to small pores), achieved a 14% improvement in filtration efficiency and a substantial 30% reduction in air resistance [73].

### 5.2. Thermal Insulation

**Figure 7:**
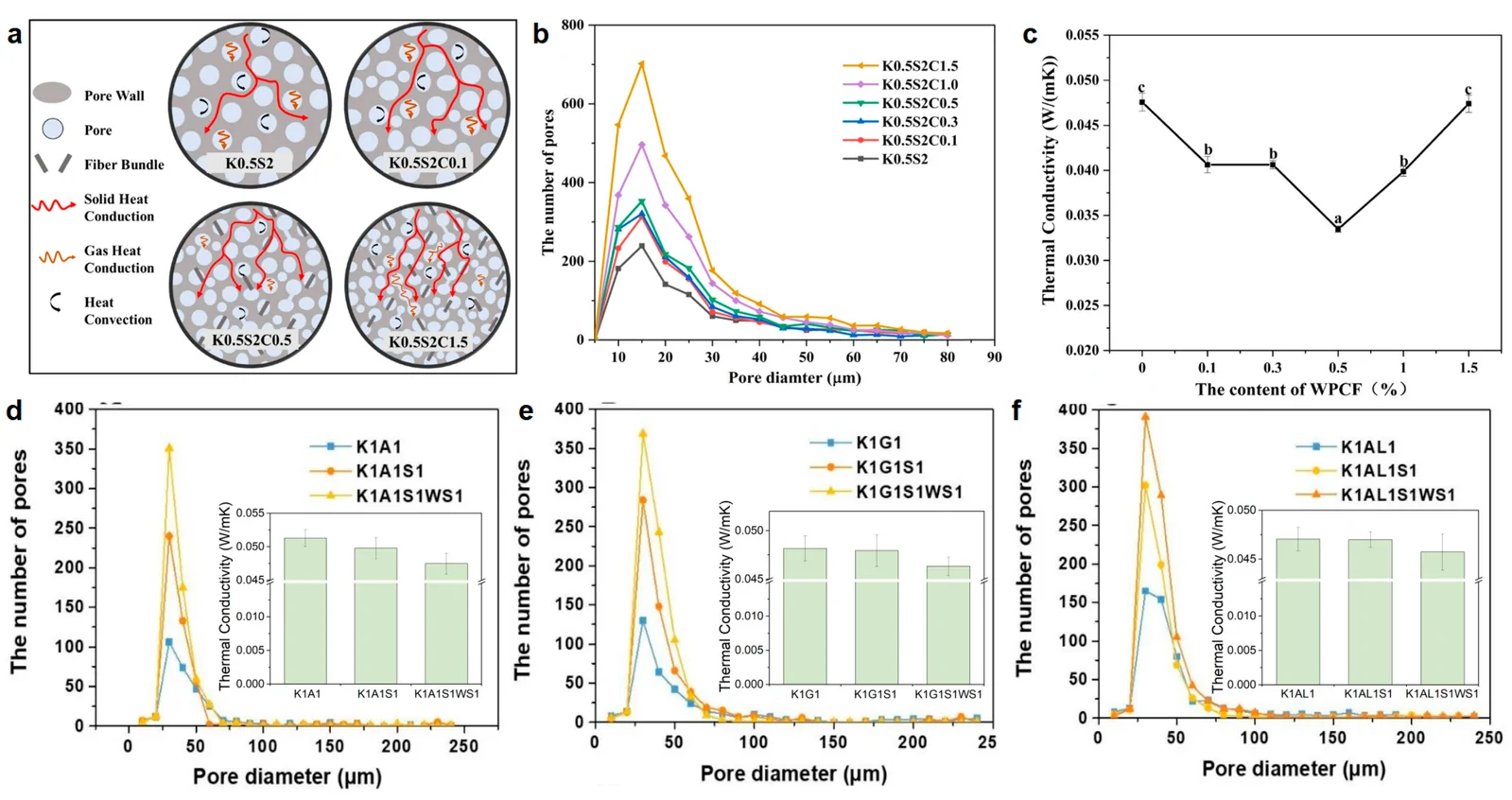
(**a**) Heat transfer mechanism of KGM-based aerogel. Lowercase letters in (**c**) indicate significant differences (*p* < 0.05). (**b**,**c**) Different pore size distributions and thermal conductivities of KGM-based aerogels [72]. Copyright 2022, Elsevier. (**d**,**e,f**) The effect of pore size distribution on the thermal conductivity of KGM-based aerogels [82]. Copyright 2021, MDPI.

The essence of heat transfer is a form of energy conversion that occurs between regions of differing temperatures. According to the second law of thermodynamics, this spontaneous heat transfer inherently moves from areas of higher temperature to those of lower temperature. Typically, heat transfer mechanisms are categorized into three types: thermal conduction, thermal convection, and thermal radiation. In aerogel materials, the porous structure significantly prolongs the path of thermal conduction. Furthermore, the pore structure consists of numerous gas–solid interfaces where substantial heat loss occurs during conduction, thereby diminishing the effect of thermal convection (Figure 7a). When the pore size of a porous material is less than 4 mm and the Grashof number (Gr) multiplied by the Prandtl number (Pr) is less than 10^3^, the influence of convective heat transfer between fluids within the pores can be neglected.

The thermal insulation performance of aerogels arises from the synergistic interplay among pore size, porosity, tortuosity, and density. When the pore size is smaller than the mean free path of gas molecules (approximately 70 nm), the Knudsen effect significantly diminishes the thermal conductivity of gases [83]. In this regime, gas molecules collide more frequently with the pore walls than with one another, leading to a suppression of energy transfer efficiency between the molecules. Consequently, the gas phase is unable to facilitate effective heat transport through molecular collisions, which markedly reduces its contribution to the overall thermal conductivity [84,85]. These nanoscale pore structures confine gas molecules within the pores, where collisions occur to dissipate heat. Due to the small pore size of aerogel materials, the internal pore walls are both continuous and complex [86]. Thermal radiation undergoes repeated reflection, scattering, or absorption on different pore walls, thereby reducing the material’s radiative heat transfer.

Pore size is a critical factor in achieving optimal thermal conductivity. The incorporation of wheat straw significantly reduces the pore size of the aerogel. Due to the unique porous structure of cellulose fiber from office waste paper (WPCF), the KGM-based aerogel exhibits low density (30.1–44.9 mg/cm^3^). With an increase in the dosage of WPCF, the total number of pores increases, leading to a greater number of small pores within the diameter range of 10–40 µm. At an optimal addition level, the thermal conductivity of the aerogel decreases to 0.0335 Wm^−1^K^−1^. (Figure 7b,c) [72]. By adjusting the amounts of starch and wheat straw, the pore size distribution of the biomass aerogel was modified. As the total number of pores increased, leading to more complex gas flow paths, the thermal conductivity of the aerogel decreased from 0.05127 W/mK to 0.04748 W/mK (Figure 7d,e).

The blocks-building conceptual framework provides a new perspective for enhancing the thermal insulation performance of biomass aerogels. Instead of focusing on reducing pore size, it aims to construct double-layer/triple-layer composite aerogel structures with lower thermal conductivity by using aerogels of different air permeability as outer layer materials, thereby reducing the air flow/thermal convection effect inside the aerogel. The results show that the triple-layer method has a more significant impact on reducing thermal conductivity compared to the double-layer approach, which is attributed to its greater effect on minimizing air flow. Additionally, the thinner the outer aerogel layer, the better. Through the triple-layer aerogel method, the thermal conductivity is significantly reduced from 0.0335 W/(m·K) to 0.0279 W/(m·K). A polysaccharide-based aerogel composed of sodium alginate, konjac glucomannan, and melamine phytate (MPA) nanosheets assembled through supramolecular interactions. Due to the excellent char-forming and non-combustible gas release properties of MPA nanosheets, the thermal stability and flame retardancy of the aerogel are significantly improved, with the limiting oxygen index reaching up to 42.4% [87].

### 5.3. Sound Absorption and Acoustic Performance

**Figure 8:**
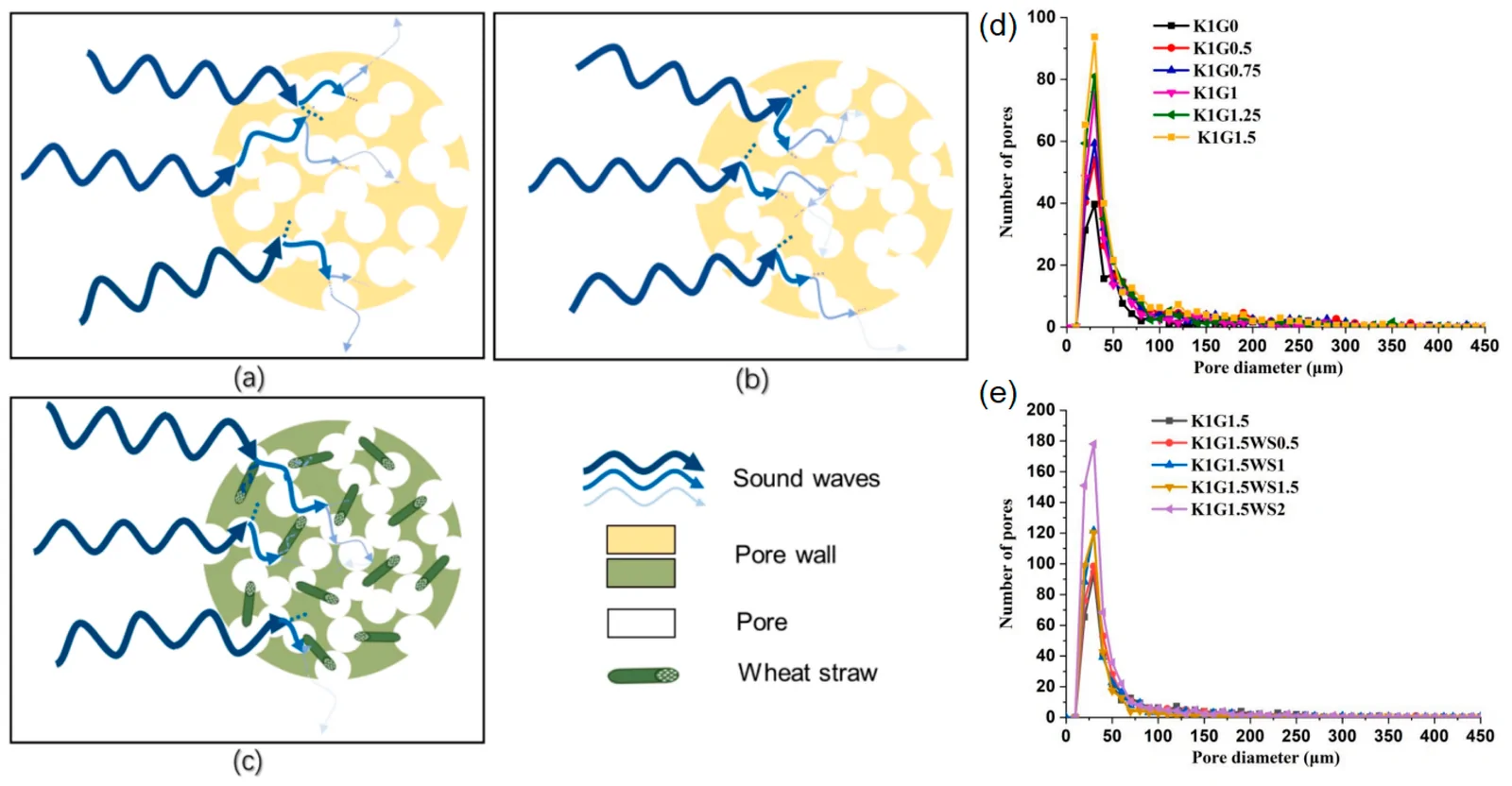
(**a**–**c**) Illustration of sound absorption by biomass aerogels with different pore structures ((**a**). K1G0.5, (**b**). K1G1.5 and (**c**). K1G1.5WS1). (**d**,**e**) Distribution of pore sizes 0–450 μm in KGM/gelatin/wheat straw aerogels with different wheat straw concentrations [91]. Copyright 2022, Elsevier.

The multiscale, highly interconnected porous network structure of biomass aerogels exhibits multiple dissipation effects on sound wave energy [88]. The acoustic performance of biomass aerogels must be evaluated with respect to two distinct mechanisms: sound absorption and sound insulation. Sound absorption refers to the dissipation of incident acoustic energy through viscous friction and thermal conduction within the porous network, while sound insulation describes the capacity to block sound transmission, which is primarily governed by mass law and structural rigidity (Figure 8a–c) [89,90].

The acoustic absorption performance of aerogels is governed by the combined effects of porosity, tortuosity, connectivity, and morphology [92,93]. Porosity is directly proportional to the volume of air available for sound wave dissipation, providing ample space for the conversion of acoustic energy into thermal energy. Tortuosity serves as a critical parameter; higher tortuosity increases the propagation path length of sound waves, thereby enhancing viscous friction and thermal dissipation. Interconnected open pore structures facilitate the deep penetration of sound waves into the material, allowing for multiple dissipation events at pore walls and fiber skeletons [94,95]. Regarding morphology, hierarchical pore structures—comprising micropores, mesopores, and macropores—can effectively broaden the sound absorption frequency band. Therefore, an optimal acoustic aerogel should exhibit high porosity, highly tortuous interconnected channels, and a well-defined hierarchical pore structure.

The multi-level porous structure significantly enhances noise reduction performance. A small amount of wheat straw was judiciously incorporated to improve sound energy dissipation through its unique cavity structure, thereby achieving a noise reduction coefficient of 0.38 (Figure 8d,e). The incorporation of gelatin significantly improves the mid-to-high frequency noise reduction performance and mechanical strength of KGM-based aerogels, achieving a noise reduction coefficient of 0.88 at 4500 Hz. This demonstrates a superior noise reduction performance when compared to melamine foam [91]. Utilizing polysaccharides such as cyclodextrin (CD), sodium alginate (Alg), and phytic acid (PA) as raw materials, PCD/Alg/PA aerogels were synthesized through freeze-drying technology. The nanoscale cavities of CDs, combined with the abundant hierarchical porous structure in PCD/Alg/PA aerogels, exhibit exceptional sound absorption capability, achieving a sound absorption coefficient exceeding 0.85 within the frequency range of 1200–6400 Hz [96]. The microstructural evolution of chitosan (CS)/polyvinyl alcohol (PVA) aerogels was examined through compositional adjustments. CS/PVA aerogels with significantly low density (0.0240 g·cm^−3^) and high porosity (98%) were successfully fabricated. The sound transmission loss (STL) exceeds 20 dB in the frequency ranges of 450 to 1000 Hz and above 3000 Hz, with a noise reduction coefficient (NRC) of 0.428 at a mere thickness of 18 mm [90].

## 6. Conclusions and Outlook

The application performance of high-performance bio-aerogels primarily depends on the precise control and design of the pore structures. Based on extensive literature on bio-aerogels, this review summarizes and proposes three theories of pore structure design that span from nano- to micro-scale: the ice crystal pore-making, the space-occupying, and the blocks-building conceptual frameworks. Among the three frameworks, ice crystal pore-making is the most established, with its freeze-drying-based ice templating mechanism extensively validated across KGM, chitosan, alginate, and starch systems. The relationships between freezing parameters and pore characteristics are well documented, providing a foundation for reliable micro-scale engineering. In contrast, several strategies remain conceptual: SAP and iodine crystals have a clear theoretical basis but lack experimental evidence for pore replication in biomass aerogels. The nanobubble approach remains speculative. Additionally, the blocks-building framework, while conceptually attractive for multifunctional design, requires further development to address interfacial adhesion and defect formation. The practice case of biomass aerogels with diverse pore structures across multiple domains is also explored. These conceptual frameworks could provide theoretical guidance on enhancing the performance and application prospects of biomass aerogels in various fields, while some challenges remain to be addressed in future work.

First, for the ice crystal pore-making conceptual framework, the precise regulation of ice crystal growth in complex multi-component precursor solutions continues to pose significant challenges. Future advancements should aim to deepen the understanding of ice crystal nucleation and growth mechanisms, while elucidating the effects of various biomass components and assembly structures on ice crystal control.

Second, for the space-occupying conceptual framework, the large-scale and controllable preparation of nanoscale, monodisperse occupying agents that exhibit high compatibility with biomass precursors remains another significant challenge. Further exploration is necessary to prevent the aggregation of occupying substances and to avoid interface damage during freezing or gelation processes, ensuring that the final pore structure aligns precisely with design expectations.

Third, scaling up the precise design of the pore structure in bio-aerogels for industrial-scale material manufacturing presents significant challenges. While techniques such as directional freezing, microfluidics, and layer-by-layer assembly facilitate controlled construction of materials at a small scale in laboratory settings, the complexities of managing temperature fields and flow fields increase exponentially as the scale is enlarged. A comprehensive understanding of the mechanisms of pore formation, grounded in pore structure design theory, is essential for developing precursor solutions with tailored rheological properties. This understanding will enable the creation of desired pore structures under more accommodating processing conditions.

Fourth, despite the promising performance of bio-aerogels at the laboratory scale, several technological and economic barriers impede their production at an industrial scale. The most significant bottleneck is the energy-intensive freeze-drying process, which constitutes a major portion of manufacturing costs and limits throughput. Alternative drying strategies, such as ambient pressure drying with solvent exchange and continuous freeze-drying systems, are being investigated to address this issue. Additionally, feedstock variability, particularly when employing agricultural or forestry residues, presents another challenge; natural biomass sources exhibit inherent compositional fluctuations that can influence gelation behavior and the reproducibility of the final pore structure. Furthermore, space-occupying strategies (e.g., SAP, iodine) encounter substantial scale-up difficulties, as maintaining uniform template dispersion and ensuring complete template removal at industrial volumes remains technically challenging. Finally, economic feasibility necessitates careful optimization of raw material selection, processing conditions, and product yield. Future efforts should focus on developing continuous, low-energy manufacturing processes and robust quality control protocols to facilitate the transition from laboratory innovations to industrial applications.

Finally, the characteristic parameters and application scope of future bio-aerogels are expected to expand significantly. Structural research will transition from merely achieving specific structures to precise design, accompanied by accurate prediction and optimization of their performance. By deeply integrating multiple disciplines, including materials science, fluid mechanics, computational simulation, and precision manufacturing, the theoretical framework for aerogel structural design will be further refined. This advancement will enhance both the performance potential and application prospects of bio-aerogels across a broader range of fields.

## Figures and Tables

**Table 1:** Comparison of applicability criteria for structural design of biomass aerogels under different conceptual frameworks.

Design Strategy	Evidence Level	Achievable Pore Size	Key Limitation	Cost	Primary Applications
Ice crystal pore-making	Experimentally demonstrated	~1–1000 µm	Hard to control < 1 µm	Moderate; freeze-drying equipment is relatively affordable; no organic solvents required	Thermal insulation, filtration, biomedical scaffolds
Space-occupying (SAP)	Indirectly supported (pore formation via ice templating in SAP-containing hydrogels demonstrated [24])	~100 nm–5 µm (estimated)	Stability of SAP particles	Moderate to high; space-occupying materials add material costs; freeze-drying still required	Nanoscale adsorption, precise molecular sieving, catalysis supports, controlled drug release
Space-occupying (Nano-sublimable substance)	Indirectly supported (iodine as pore-making agent demonstrated in porous carbon [25])	~100 nm–1 µm (estimated)	Requires sublimation control		
Space-occupying (Nano bubbles)	Indirectly supported (constructing hierarchical pores within cryogels [26])	~10 nm–1 µm (estimated)	Prevent bubbles from bursting		
Blocks building	Indirectly supported	Multi-scale	Interfacial defects	Moderate; additional assembly steps increase processing cost; material costs depend on unit composition	Multi-functional integrated systems (e.g., gradient filtration + adsorption + catalysis)

**Table 2:** Effects of different freezing conditions on the pore structure of biomass aerogels.

Freezing Condition	Biomass Type	Pore Structure	Mechanism	References
Freezing direction	Cellulose	The bidirectionally freeze-dried aerogels showed a larger specific surface area and a narrower pore size distribution.	The bidirectionally freeze-dried aerogel applied a temperature gradient in both directions, leading to ice nucleation at the bottom, with faster cooling at the liquid nitrogen end.	[35]
	Bacterial cellulose	Aerogels prepared by the bidirectional freezing technique showed a bridge-like structure with connecting neighboring laminae.	Compared with disordered and unidirectional freezing, bidirectional freezing can assemble efficiently in multiple dimensions and results in long-range ordered structures	[36]
	Alginate/cellulose	The total pore area reached 20.542 m^2^·g^−1^ and enhanced the pore connectivity	Bidirectional freeze-drying technology can effectively induce the directional growth of ice crystals by introducing a temperature gradient in two directions	[37]
Freezing refrigerant	Soy protein	A196 aerogel exhibited a uniform and dense needle-like structure with the smallest pore size	Unlike air-based freezing at higher temperatures, freezing in liquid nitrogen (−196 °C) led to an extremely rapid formation of ice nuclei, resulting in the growth of numerous small ice crystals	[38]
Freezing temperature	Konjac glucomannan	With decreased pre-freezing temperature, aerogel pores went smaller, but finally exhibited a dendritic structure	At a lower pre-freezing temperature, a larger undercooling led to an increased rate of nucleation of ice crystals, and the ice crystal growth rate was less than the nucleation rate, contributing to a large number of small ice crystals	[33]
	Xanthan gum	Aerogel frozen at −40 °C displayed an ordered hierarchical thin sheet structure, forming a network composed of co-aligned and pore channels is formed	Low temperature promoted the formation of small ice crystals	[39]

## Data Availability

No new data were created or analyzed in this study. Data sharing is not applicable to this article.
